# Do community-based strategies reduce HIV risk among people who inject drugs in China? A quasi-experimental study in Yunnan and Guangxi provinces

**DOI:** 10.1186/1477-7517-11-15

**Published:** 2014-05-06

**Authors:** Kai Wang, Hongyun Fu, Kim Longfield, Shilpa Modi, Gary Mundy, Rebecca Firestone

**Affiliations:** 1 PSI/China, 909-9 F, M2 Building, Harmonious Society, Xiaokang Rd, Hongyun Community, Wuhua District, Kunming 650000, China; 2 PSI/China, 12711 Misty Creek Drive, Little Rock, AR 72211, USA; 3 PSI, 1120 19th Street NW, Suite 600, Washington, DC 20036, USA; 4 PSI Asia Region, Unit 12A, 12th Floor, Q House Convent Building, 38 Convent Road, Silom, Bangrak, Bangkok 10500, Thailand

**Keywords:** Social marketing, Drop-in center, Community-based outreach, Behavior change communication (BCC), People who inject drugs (PWID), Respondent-driven sampling (RDS), Needle-syringe exchange program (NSP), Condom use, HIV testing and counseling (HTC), Coarsened exact matching (CEM)

## Abstract

**Background:**

HIV transmission among people who inject drugs (PWID) is high in Yunnan and Guangxi provinces in southwest China. To address this epidemic, Population Services International (PSI) and four cooperating agencies implemented a comprehensive harm reduction model delivered through community-based drop-incenters (DiC) and peer-led outreach to reduce HIV risk among PWID.

**Methods:**

We used 2012 behavioral survey data to evaluate the effectiveness of this model for achieving changes in HIV risk, including never sharing needles or syringes, always keeping a clean needle on hand, HIV testing and counseling (HTC), and consistent condom use. We used respondent-driven sampling to recruit respondents. We then used coarsened exact matching (CEM) to match respondents during analysis to improve estimation of the effects of exposure to both DiC and outreach, only DiC, and only outreach, modeled using multivariable logistic regression.

**Results:**

We found a significant relationship between participating in both peer-led DiC-based activities and outreach and having a new needle on hand (odds ratio (OR) 1.53, *p* < .05) and consistent condom use (OR 3.31, *p* < .001). We also found a significant relationship between exposure to DiC activities and outreach and HIV testing in Kunming (OR 2.92, *p* < .01) and exposure to peer-led outreach and HIV testing through referrals in Gejiu, Nanning, and Luzhai (OR 3.63, *p* < .05).

**Conclusions:**

A comprehensive harm reduction model delivered through peer-led and community-based strategies reduced HIV risk among PWID in China. Both DiC activities and outreach were effective in providing PWID behavior change communications (BCC) and HTC. HTC is best offered in settings like DiCs, where there is privacy for testing and receiving results. Outreach coverage was low, especially in Guangxi province where the implementation model required building the technical capacity of government partners and grassroot organizations. Outreach appears to be most effective for referring PWID into HTC, especially when DiC-based HTC is not available and increasing awareness of DiCs where PWID can receive more intensive BCC interventions.

## Background

In 1989, China reported its first cases of HIV among 146 heroin users in Yunnan Province, along China's southwest border [1]. Yunnan is located near the Golden Triangle, consisting of Thailand, Myanmar, and Laos. The Golden Triangle is one of the three largest heroin production sites in the world. As a result, HIV spread quickly along drug trafficking routes in Yunnan and into neighboring provinces [2].

According to an assessment by the World Health Organization, Joint United Nations Programme on HIV/AIDS, and the Chinese Ministry of Health, China's overall HIV prevalence has remained relatively low between 0.5%–0.6% in 2011 [3,4]. However, the national prevalence of HIV among people who inject drugs (PWID) was 9.08% in 2010, with prevalence higher than 50% in parts of southwest China [3,4]. The provinces of Yunnan and Guangxi have two of the highest concentrations of HIV cases, as well as registered drug users in China. Yunnan and Guangxi account for 22% of new HIV cases in the country, despite making up only 6.9% of the national population [5]. In Guangxi, there are approximately 50,000 registered PWID, with injecting drug use accounting for over 69% of the cumulative reported cases of HIV in the province [6].

Transmission of HIV through unsafe injecting practices, including sharing of needles and syringes, has remained high since the early stages of the epidemic in China. At the national level, prevalence of ever sharing injecting equipment among PWID was 25% in 2011 [7].Of the 780,000 people in China estimated to be living with HIV (PLHIV) in 2011, 28.4% were infected through injecting drug use [2].

In 2004, in response to the growing HIV epidemic, China implemented methadone maintenance therapy (MMT), needle-syringe exchange programs, and condom promotion at the national and local levels [2]. Prior to this, China's approach to working with PWID was largely punitive. Drug users were sent to compulsory detoxification programs and re-education through labor (RTL) centers [8]. In 2008, China passed the Narcotic Control Law, which supports community-based rehabilitation for PWID rather than sending PWID to compulsory detoxification programs and RTL centers [9]. Although recent policies have created a more supportive environment for PWID, a number of barriers still exist for PWID trying to access HIV testing and counseling (HTC) and harm reduction services, including those for preventing sexual transmission of HIV [10,11]. These barriers include community stigma and discrimination, criminalization of illicit drug use, and low coverage of existing programs [9].

Despite the establishment of a more supportive environment, limited evidence is available on the ability of the current network of harm reduction and HIV prevention strategies to improve HIV risk behaviors. Evaluations of these programs have examined process measures for service quality and have demonstrated expanded population coverage [10-12], and recent evidence is available that HIV and hepatitis B incidence may decline among PWID enrolled in multipronged harm reduction programs [2]. More direct evidence on program effectiveness and a link to behavior change is still needed.

### The comprehensive prevention package in southwestern China

**Figure 1:**
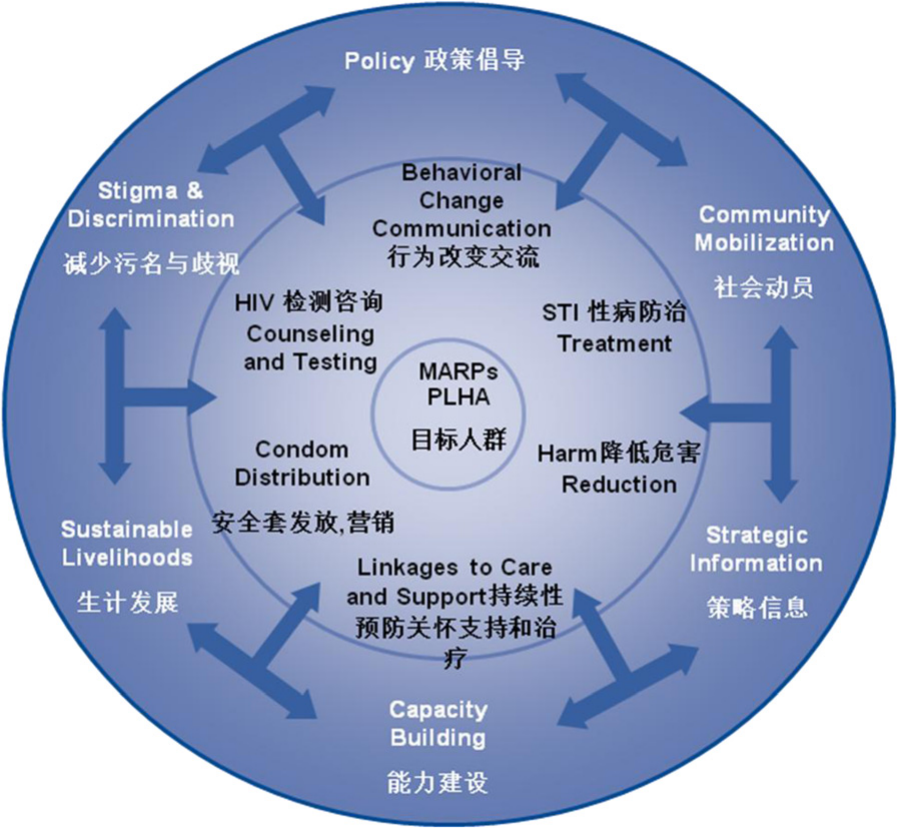
USAID comprehensive prevention package (CPP) model.

Since 2009, Population Services International (PSI) has implemented a comprehensive prevention package (CPP) for PWID in Yunnan and Guangxi provinces, in collaboration with a consortium of partners, including FHI 360, the International HIV/AIDS Alliance, Research Triangle Institute, Pact, and local government partners with support from the United States Agency for International Development (USAID) [13,14]. The CPP program is a community-based strategy, delivered through local partners, that helps PWID access and use a core set of interventions to reduce HIV infection. In Yunnan and Guangxi, PWID access CPP interventions through drop-in centers (DiC) and outreach implemented by PSI's peer educators, PSI-supported community-based organizations (CBOs), and other local partners. The following set of CPP interventions (Figure 1) was provided through DiC and outreach: (1) peer-led behavior change communications and education to promote condom use and prevent STIs, HIV, hepatitis B and C, and tuberculosis; (2) provision of condoms and lubricants; (3) referrals for anti-retroviral therapy (ARV) treatment, MMT, and community rehabilitation; (4) needle-syringe exchange and education regarding safe injecting practices and overdose prevention; and (5) promotion of ARV and MMT adherence. Additionally, project partners conducted a set of essential ‘enabling environment’ interventions that aimed to improve policies through advocacy, reduce stigma and discrimination, support community mobilization, build capacity of local organizations, support income generation for PWID, and use strategic information for decision-making.

In Yunnan province, PSI implemented the Huxianghao (HXH, ‘Good for you good for me’) program in Kunming city, which included DiC and outreach activities to deliver a wide range of services for PWID. PSI also promoted the replication of the established HXH program model in Kunming through technical assistance (TA) to community rehabilitation programs. In Gejiu city, PSI and project partners provided TA and funding support to PWID-led community-based organizations to conduct DiC and outreach activities.

In Guangxi province, PSI-implemented outreach activities in Nanning city and provided TA to the local CDC, community rehabilitation programs, and community-based organizations in Nanning and Luzhai City to conduct DiC and outreach activities. Project partners also supported community-based organizations by working on MMT and ARV adherence with PWID and with the Luzhai CDC to conduct outreach. In Yunnan, the DiC and outreach activities included HTC, whereas in Guangxi, clients were referred to hospital or government centers for testing.

### Objective

Following several years of program implementation, we aimed to evaluate the effectiveness of this comprehensive harm reduction model delivered through participation in community-based DiCs and interpersonal outreach for improving HIV risk behavior among PWID in southwestern China. Given the nature of the program, we considered harm reduction practices (never sharing needles or syringes, always keeping a clean needle on hand), consistent condom use with different types of sexual partners (including regular, casual, and commercial partners), and use of HTC. Our evaluation is not designed to compare implementation models but rather assess the effect of the overall package of CPP interventions on HIV risk among PWID.

## Methods

### Study population and sampling

From March to April 2012, we used respondent-driven sampling (RDS) to recruit 1,035 PWID from four cities in Yunnan and Guangxi provinces for a behavioral survey. Sample sizes were as follows: Kunming (*n* = 336) and Gejiu (*n* = 204) in Yunnan and Nanning (*n* = 355) and Luzhai (*n* = 140) in Guangxi. RDS is a chain-referral sampling method used to recruit hard-to-reach populations, including PWID [15-17]. RDS relies on the assumption that, given sufficiently long referral chains (i.e., 3–6 waves of respondents), the sample composition becomes stable or reaches ‘equilibrium’, which results in a sample that has the characteristics of a probability sample. We then used sampling weights to account for the social networks used in chain referral recruiting.

We started by recruiting 4–8 seeds in each study city. We gave each seed three coupons and asked him/her to recruit three peers from his/her social network to participate in the study. We diversified seeds based on age, sex, exposure to program interventions, current MMT use, and current injecting status (current or former PWID). The recruitment coupons were uniquely numbered, which allowed us to link seeds to their referrals and track the length of recruitment chains. Participants included in the study were those who were aged 18 to 49, intravenous drug users in the past 12 months, residing in one of the study cities for a minimum of 1 month, able to speak and comprehend Mandarin Chinese enough to respond to survey questions, and not under the influence of drugs or alcohol at the time of the survey.

We collected data through face-to-face interviews in locations where respondents' privacy could be protected. The questionnaire covered several topics, including demographic characteristics, injecting behaviors, sexual activity, condom use, STI testing, HIV testing, and exposure to program activities. The Yunnan Institution for Drug Abuse Institutional Review Board provided ethical review and approval. We obtained informed consent before conducting interviews. All interviewers and others associated with the study completed training on human subjects' protection.

### Measures

We assessed four outcomes of interest, all treated as binary variables: not sharing needles or syringes in the past 3 months, keeping a new needle on hand in the past 3 months, having received an HIV test in the past 12 months, and consistent condom use with any type of sexual partner in the past 3 months. We created several variables to assess exposure to the program model through its different delivery channels: participation in any program activity whether DiC-based or outreach in the past 12 months, participation in both DiC activities and outreach in the past 12 months, participation in only DiC activities in the past 12 months, and participation in only outreach in the past 12 months. Each variable was coded as a dichotomous measure, indicating that some respondents were not exposed to any program channel. Other variables within the analysis included: age, sex, working hours, city of residence, ever use of MMT, ethnicity (Han Chinese or not), education (high school and higher vs. less than high school), and number of sex acts in the past 3 months.

### Analysis

This analysis was intended to be representative of PWID in Yunnan and Guangxi. We used RDSAT to estimate sampling weights within each city [18]. We then pooled the data across all cities and calculated weights for each city based on population size estimates provided by project partners. These weights were used in all subsequent analyses.

Because we aimed to test the effectiveness of the CPP model, we next used a matching technique, coarsened exact matching (CEM), to create statistically equivalent groups of exposed and nonexposed respondents. This quasi-experimental approach allowed us to designate a counterfactual (no participation in either DiC or outreach activities) when an experimental design was not feasible, given that program implementation was ongoing and was not designed or implemented with intervention and control populations [19-21]. Coarsened exact matching is a monotonic imbalance matching method designed to reduce imbalance between treatment and control groups in observational data [22]. CEM assigns each case into one of a specified set of strata in which members are exactly matched on a set of coarsened, or categorized, variables. Matched cases are then assigned a weight specific to that stratum and representative of the proportion of all cases present in the stratum [19-21,23]. We chose CEM over other matching techniques, such as propensity score matching, to achieve balanced groups, reduce the need for multiple iterations and re-matching, and maximize the number of possible matches in our sample [22-24].

In the pooled dataset, we matched survey respondents on age (continuous variable coarsened to <40 years, ≥40), sex, working hours (coded as end work before 5 p.m./start work after 5 p.m./not working vs. all other working hours), resident city, and ever used MMT. We selected these five variables after consultation with program staff because they were identified as having a substantial influence on PWID's likelihood of participation in the interventions. Controls were thus defined as people who did not participate in either DiC or outreach, but who were identified, through matching, as having a similar probability of participation as those who participated in any program activity. During routine data collection for the program, we found that older people were more likely to use DiC services than youths. Peer educators reported that males were more likely than females to use DiCs and MMT services. Respondents' working hours could affect their ability to visit DiCs during opening hours or participate in outreach. We matched on resident city account for variations in the political environmental towards drug use and harm reduction in each city. Since peer educators conducted outreach in MMT centers, PWID who ever used MMT services would have had greater opportunity to access outreach and know about DiCs. This match yielded an L1 distance of 2.83^E−16^, indicating that the matched subsample had minimal imbalance between those exposed and not exposed to the program.

We calculated descriptive statistics for both the full, pooled dataset, and the matched sample. We applied combined RDS and city weights to analyze pooled data. For analysis of the matched subsample, we applied one combined weight derived from CEM weights, RDS weights, and city weights. We then created bivariate and multivariate logistic regression models, controlling for ethnicity and education. After assessing the effects of any program participation (not shown), we tested the isolated effects of participation in both DiC and outreach, participation in DiC only and participation in outreach only. Separate logistic regression models were constructed for each treatment variable to isolate the effects of each channel. All analyses were conducted in Stata 11.

## Results

### Population characteristics

Table 1 shows the pooled and matched estimate for population characteristics; we only discuss matched results henceforth. The majority of respondents were male (67.4%), over 40 years old (51.6%), of Han ethnicity (83.3%), and had less than a high school education (68.9%). Only 31.1% of respondents were married: the majority had never been married or were widowed or divorced. The use of MMT was very high: nearly 88% of respondents had received MMT sometime in the past.

**Table 1:** Population characteristics of people who inject drugs in four southwestern Chinese cities in 2012

**Population characteristics**	**Pooled sample**	**Matched Sample**
	**(*n*** **= 1,035) (%)**	**(*n*** **= 975) (%)**
Sex (male)	64.0	67.4
Age (in years)		
18 to 30	4.8	7.2
31 to 40	32.9	41.2
41 to 49	62.3	51.6
Ethnicity (Han)	87.3	83.3
Education (High school education or above)	32.5	31.1
Marital status		
Never married	38.4	42.6
Currently married	29.9	31.1
Divorced or widowed	31.7	26.3
Ever received methadone maintenance treatment (MMT)	84.7	87.9

### Behavioral outcomes and exposure to DiC and outreach

We present behavioral outcomes and program exposure in Table 2. More than 80% of respondents had injected heroin in the past 3 months. Reported rates for needle and syringe sharing were only 6.2%, much lower than rates reported in the literature. However, less than a third (32.3%) of respondents reported keeping clean needles on hand. The majority of the respondents reported having an HIV test during the past year (71.6%). More than half of the respondents reported having sex in the past 3 months (54.6%), with an average of 13 sex acts over the course of 3 months. Among those who were sexually active, 49.3% reported consistent condom use with all partners during the same period.

**Table 2:** Behavioral outcomes and exposure to drop-in center and outreach activities

	**Kunming (*n*** **= 336)**	**Gejiu (*n*** **= 204)**	**Nanning (*n*** **= 355)**	**Luzhai (*n*** **= 140)**	**Pooled sample (*n*** **= 1,035)**	**Matched Sample (*n*** **= 975)**
	**%**	**%**	**%**	**%**	**%**	**%**
Behavioral outcomes						
Injected heroin in the past 3 months	72.6	81.4	83.9	81.4	75.8	80.2
Ever shared needles/syringes with others in the past 3 months (among those who used heroin in the past 3 months)	5.0	0.6	8.6	10.0	5.2	6.2
	(*n* = 244)	(*n* = 166)	(*n* = 298)	(*n* = 114)	(*n* = 822)	(*n* = 762)
Always kept a new needle on hand during the past 3 months (among those who used heroin in the past 3 months)	26.2	38.8	24.8	29.4	28.0	32.3
Received an HIV test in the past 12 months	67.6	75.9	52.1	66.5	66.5	71.6
Had sexual intercourse in the past 3 months	61.6	46.1	52.4	55.0	57.8	54.6
Mean number of sex acts in the past 3 months	13	10	17	19	14	13
	(*n* = 207)	(*n* = 94)	(*n* = 186)	(*n* = 77)	(*n* = 564)	(*n* = 500)
Used condoms consistently with all partners in the past 3 months (among those who had sexual intercourse in the past 3 months)	38.5	67.0	30.2	45.6	41.4	49.3
	(*n* = 207)	(*n* = 94)	(*n* = 186)	(*n* = 77)	(*n* = 564)	(*n* = 500)
Program exposure						
Participated in either DiC activities or outreach in the past 12 months	58.3	78.4	25.6	51.4	55.9	51.0
Participated in DiC activities and outreach in the past 12 months	25.9	25.0	9.9	10.0	22.8	18.4
Participated in DiC activities only in the past 12 months	22.0	50.5	7.0	38.6	24.2	24.9
Participated in outreach only in the past 12 months	10.4	2.9	8.7	2.9	8.9	7.7

Just over half of the respondents reported attending either DiC-based activities or receiving outreach during the past 12 months (51.0%). Participation in DiC-based activities was higher than outreach (43.3% vs. 26.1%).About one fifth of the respondents reported attending both DiC-based activities and receiving outreach during the past 12 months (18.4%). Exposure to DiC activities was the highest in Gejiu (75.5%), approximately 48% for both Luzhai and Kunming, and much lower in Nanning (16.9%). Exposure to outreach was highest in Kunming (36.3%), followed by Gejiu (27.9%), Nanning (18.6%), and Luzhai (12.9%). CPP staff report that low levels of outreach in Nanning are explained by the size of the city and that there are few qualified outreach workers to reach the PWID community. The model for outreach is also different in Guangxi than in Kunming; CPP staff focused more on TA to build the capacity of community rehabilitation centers, government partners, and grassroot organizations to conduct outreach. This is a slower model to roll out because on-the-job training is intensive, and it requires a great deal of supervision. The outreach model in Guangxi also required more consensus building and agreement on program goals than the Kunming model, which takes time and can slow down program execution. The lack of correlation between exposure to outreach and some of the behavioral outcomes may be attributable to low levels of coverage in places like Nanning and Luzhai.

Tables 3, 4, and 5 present results for the pooled and matched samples and show the correlation between program exposure and the behavioral outcomes. We present bivariate and multivariate analyses to demonstrate how the models were constructed, but we only focus on the matched multivariate analyses here.

**Table 3:** Effectiveness of exposure to drop-in center and outreach activities on safer injecting practices

	**Never shared needles or syringes in the past 3 months**				**Always kept a new needle on hand in the past 3 months**			
**Program exposure**	**Pooled sample (*n*** **= 823)**		**Matched sample (*n*** **= 762)**		**Pooled sample (*n*** **= 823)**		**Matched sample (*n*** **= 762)**	
	**Bivariate**	**Multivariate**	**Bivariate**	**Multivariate**	**Bivariate**	**Multivariate**	**Bivariate**	**Multivariate**
	**OR (95% CI)**	**Adjusted OR (95% CI)**	**OR (95% CI)**	**Adjusted OR (95% CI)**	**OR (95% CI)**	**Adjusted OR (95% CI)**	**OR (95% CI)**	**Adjusted OR (95% CI)**
Participated in DiC activities and outreach in the past 12 months	1.37 (0.61 to 3.09)	1.32 (0.59 to 2.99)	1.41 (0.58 to 3.42)	1.40 (0.57 to 3.40)	1.50* (1.05 to 2.14)	1.49* (1.05 to 2.14)	1.53* (1.07 to 2.19)	1.53* (1.07 to 2.19)
Participated in DiC activities only in the past 12 months	0.80 (0.40 to 1.61)	0.80 (0.40 to 1.62)	0.53 (0.26 to 1.09)	0.55 (0.27 to 1.13)	1.01 (0.71 to 1.45)	1.03 (0.71 to 1.47)	1.27 (0.87 to 1.83)	1.28 (0.88 to 1.85)
Participated in outreach only in the past 12 months	0.53 (0.22 to 1.33)	0.56 (0.22 to 1.39)	0.48 (0.19 to 1.23)	0.53 (0.21 to 1.37)	1.07 (0.62 to 1.85)	1.08 (0.62 to 1.88)	1.03 (0.59 to 1.79)	1.06 (0.61 to 1.86)

*Significant at *p* < 0.05. Age, gender, respondents' working hours, resident city, and ever use of MMT were matched for the CEM model. Covariates were ethnicity and education in the separated logistic regression models but not reported here.

**Table 4:** Effectiveness of exposure to drop-in center and outreach activities on HIV testing

	**Tested for HIV in the past 12 months**				**Tested for HIV in the past 12 months**			
	**(Kunming)**				**(Gejiu, Nanning, and Luzhai)**			
**Program exposure**	**Pooled sample (*n*** **= 335)**		**Matched sample (*n*** **= 324)**		**Pooled sample (*n*** **= 699)**		**Matched sample (*n*** **= 650)**	
	**Bivariate**	**Multivariate**	**Bivariate**	**Multivariate**	**Bivariate**	**Multivariate**	**Bivariate**	**Multivariate**
	**OR (95% CI)**	**Adjusted OR (95% CI)**	**OR (95% CI)**	**Adjusted OR (95% CI)**	**OR (95% CI)**	**Adjusted OR (95% CI)**	**OR (95% CI)**	**Adjusted OR (95% CI)**
Participated in DiC activities and outreach in the past 12 months	3.51*** (1.80 to 6.85)	3.50*** (1.79 to 6.86)	2.79** (1.42 to 5.47)	2.92** (1.47 to 5.78)	2.96*** (1.74 to 5.01)	2.74*** (1.67 to 4.86)	1.82* (1.06 to 3.14)	1.73 (1.00 to 2.99)
Participated in DiC activities only in the past 12 months	1.44 (0.82 to 2.54)	1.48 (0.84 to 2.62)	1.08 (0.61 to 1.93)	1.10 (0.62 to 1.98)	2.03*** (1.39 to 2.95)	2.17*** (1.48 to 3.19)	1.05 (0.71 to 1.56)	1.10 (0.73 to 1.62)
Participated in outreach only in the past 12 months	0.53 (0.27 to 1.06)	0.56 (0.28 to 1.12)	0.44* (0.22 to 0.88)	0.47* (0.23 to 0.95)	5.80** (1.88 to 17.86)	5.78** (1.87 to 17.89)	3.64* (1.18 to 11.28)	3.63* (1.17 to 11.28)

*Significant at *p* < 0.05; **significant at *p* < 0.01; ***significant at *p* < 0.001. Age, gender, respondents' working hours, resident city, and ever use of MMT were matched for the CEM model. Covariates were ethnicity and education in the separated logistic regression models but not reported here.

**Table 5:** Effectiveness of exposure to drop-in center and outreach activities on consistent condom use

	**Consistent condom use with all partners in the past 3 months**			
**Program exposure**	**Pooled sample (*n*** **= 564)**		**Matched sample (*n*** **= 500)**	
	**Bivariate**	**Multivariate**	**Bivariate**	**Multivariate**
	**OR (95% CI)**	**Adjusted OR (95% CI)**	**OR (95% CI)**	**Adjusted OR (95% CI)**
Participated in DiC activities and outreach in the past 12 months	2.25*** (1.53 to 3.31)	3.32*** (1.55 to 3.48)	2.00*** (1.35 to 2.97)	3.31*** (1.52 to 3.50)
Participated in DiC activities only in the past 12 months	1.47* (1.02 to 2.14)	1.43 (0.98 to 2.10)	1.58 (1.06 to 2.33)	1.65* (1.10 to 2.47)
Participated in outreach only in the past 12 months	0.76 (0.42 to 1.39)	0.86 (0.46 to 1.60)	0.83 (0.43 to 1.58)	0.93 (0.48 to 1.80)

*Significant at *p* < 0.05; ***significant at *p* < 0.001. Age, gender, respondents' working hours, resident city, and ever use of MMT were matched for the CEM model. Covariates were ethnicity and education in the separated logistic regression models but not reported here.

### Effectiveness of DiC and outreach on safer injecting practices

Table 3 presents associations between program exposure and safer injecting practices. There was a statistically significant relationship between participating in both DiC-based activities and outreach and having a new needle on hand (odds ratio (OR) 1.53, *p* < .05). The relationship between exposure to only DiC or only outreach and either of the two safer injecting practices was not statistically significant. There was also no relationship between both DiC-based activities and outreach and needle or syringe sharing, which was not surprising given the low levels of reported overall sharing (6.2%).

There are several contextual factors that help explain these findings. The positive relationship between exposure to both DiC-related activities and outreach and having new injecting equipment on hand is likely due to behavior change communication (BCC) in DiCs and outreach, especially in 2011 and 2012. There were also needle-syringe exchange programs in the Kunming and Geiju DiCs and the Nanning CDC, which means that in some cases, BCC is supported by having new injecting equipment to give to PWIDs.

### Effectiveness of DiC and outreach on HIV testing

Table 4 presents associations between program exposure and HIV testing. Because the model for testing was different in the intervention sites, we segmented the analysis by Kunming and the other three cities. In Kunming, the program has offered rapid HIV testing in the DiC since 2005 and through outreach since 2009. In both settings, test results are available in 10 min. In this analysis, we only found a statistically significant relationship between exposure to both DiC-related activities and outreach and testing (OR 2.92, *p* < .01). There was no association between exposure to only DiC in Kunming and testing. There was a negative association between exposure to only outreach in Kunming and testing. An explanation for this may be that if referrals from outreach to DIC were specifically for testing, then those that did not attend the DIC may have made a decision not to test. Similarly, those that used the DIC but without referral from an outreach worker may have used the facility for services other than testing. Those that used the DIC as a result of outreach contact, however, may have done so specifically to be tested for HIV, having been referred to the service by the outreach worker.

In Gejiu, Nanning, and Luzhai, only one DiC in Gejiu offered HIV testing. No immediate HIV testing was offered during outreach activities; referrals were given for testing in hospitals and government testing sites. We found a statistically significant relationship between exposure to only outreach and testing in these three cities (OR 3.63, *p* < .05). This model of only outreach with referrals for testing appears to be working and an appropriate strategy when testing is not available in DiCs or in areas where there are no DiCs. However, participation in DiC-based activities in Gejiu, Nanning, and Luzhai was not associated with referrals for testing, which suggests that DiC staff did not sufficiently refer PWID to public hospitals or government testing sites.

### Effectiveness of DiC and outreach on consistent condom use

Table 5 shows associations between program exposure and consistent condom use. There was a statistically significant relationship between participating both DiC activities and outreach and consistent condom use (OR 3.31, *p* < 0.001), and a statistically significant relationship between participating in only DiC-based activities and consistent condom use (OR 1.65, *p* < .05), likely explained by the factors noted previously: intensive BCC and the option of speaking with CPP staff in a private setting. There was no association between exposure to only outreach and consistent condom use. These findings indicate that there is a subset of PWID at increased risk for HIV infection through unprotected sex.

These findings are in line with evidence from elsewhere in China that comprehensive, community-based harm reduction programs can contribute to HIV prevention when designed with the needs of PWID in mind. Social marketing of needles in Guangdong and Guangxi has been demonstrated to increase access to clean needles and safe injection practices [25]. HIV prevalence along the China-Vietnam border declined in the context of peer outreach and distribution of safe injection equipment [26]. Similarly, HIV and hepatitis B incidences were found to be on the decline among a cohort of PWID in Sichuan providence who received community-based harm reduction programs [2]. Findings from this study suggest how delivery channels for a comprehensive program can influence just not injecting practices but the use of HTC along with condoms to prevent sexual transmission.

### Limitations

Our study has several limitations. Study respondents self-reported HIV risk behaviors, and results are subject to social desirability bias. A limitation of RDS was that it was not possible to identify the nonresponse rate in the sample [17,27,28]. For example, we do not know how many attempts were made before three coupons were distributed successfully within PWID social networks. If particular profiles of PWID were more likely to refuse study participation, there may have been sampling bias. The other limitation of RDS is that although it has features of probability sampling, we could not estimate the extent to which it was truly representative of PWID across the four study cities. However, we calculated and applied city weights to improve representativeness of the sample. All successful recruitment waves were between 5 and 12, which suggests that the study achieved equilibrium [15,29].

A further limitation is that we may not have fully accounted for factors that influenced selection into the CPP program. We attempted to address risks of selection bias through matching on age, sex, working hours, city residence, and use of MMT. However, these findings still face the possibility of omitted variable bias. We were not able to account for other factors such as distance to DiCs or individual propensities to participate in health-promoting activities. Finally, with the matching technique we used, we may have increased the number of pairs to be matched at the expense of less exact matching. This is less of a concern with categorical variables than continuous variables. We coarsened continuous variables based on programmatically meaningful categories, and diagnostics of the matching procedure indicated a high quality match.

## Conclusion

Our analysis demonstrates that comprehensive peer-led programs reduced HIV risk among PWID. Overall, both DiC-based activities and outreach were successful at increasing safe injecting practices, HTC, and consistent condom use. Outreach coverage was low, especially in Guangxi province where the implementation model required building the technical capacity of government partners and grassroots organizations.

The findings raise questions about the independent and combined effects of different intervention activities for addressing HIV risk behaviors among PWID. While a combination of outreach and DIC was more commonly found to have an association with reduced risk behaviors, it was notable that, in areas where DICs did not offer testing services, outreach alone was strongly associated with an increased likelihood of testing. This was, presumably, a result of referral to local testing services that were not available at DICs. This suggests that outreach in itself may have an important role to play in increasing testing behavior when accessible testing services are available, regardless of whether those services are themselves ‘community based’, i.e., run from drop-in centers or other locations providing testing targeted at PWID. These strategies require further expansion to fully address the needs of PWID in southwestern China.

## Competing interests

The authors declare that they have no competing interests.

## Authors’ contributions

KW designed the study, managed fieldwork, analyzed data and created tables, and wrote portions of the manuscript. HY designed the study, analyzed data, and helped outline the manuscript. KL and SM wrote and edited the manuscript. GM helped with study design, data analysis, and reviewed the manuscript. RF helped with data analysis and wrote sections of the manuscript. All authors read and approved the final manuscript.
